# Rhegmatogenous retinal detachment following intravitreal ocriplasmin

**DOI:** 10.1007/s00417-016-3398-7

**Published:** 2016-06-08

**Authors:** Haifa A. Madi, Richard J. Haynes, Diana Depla, Morten D. de la Cour, Sarit Lesnik-Oberstein, Mahi M. K. Muqit, Niall Patton, Nick Price, David H.W. Steel

**Affiliations:** 1 Sunderland Eye Infirmary, Queen Alexandra Road, Sunderland, SR2 9HP UK; 2 Bristol Eye Hospital, Lower Maudlin Street, Bristol, UK; 3 Ophthalmology Department, Cumberland Infirmary, Carlisle, UK; 4 Eye Department, Rigshospitalet, Glostrup, Ndr. Ringvej 57, 2600 Glostrup, Denmark; 5 Department of Ophthalmology, Academic Medical Centre, Meibergdreef 9, 1100 DD Amsterdam, The Netherlands; 6 Vitreoretinal Service, Moorfields Eye Hospital, City Road, London, UK; 7 Manchester Royal Eye Hospital, Oxford Road, Manchester, UK; 8 Royal Wolverhampton NHS Trust, Wolverhampton & Midland Counties Eye Infirmary, Wolverhampton, UK; 9 Institute of Genetic Medicine, Newcastle University, Newcastle Upon Tyne, UK

**Keywords:** Macular hole, Ocriplasmin, Pharmacological vitreolysis, Retinal detachment, Retinal tears, Vitreomacular traction

## Abstract

**Purpose:**

To describe the characteristics and outcomes of patients presenting with rhegmatogenous retinal detachment (RRD) after ocriplasmin (OCP) injection.

**Methods:**

Retrospective, multi-centre, observational case series with case note review.

**Results:**

Eight patients with symptomatic vitreomacular traction (six with concomitant macular hole) were diagnosed with RRD after a median of 16 days (range 3–131 days) post-OCP injection. Presentation was within 3 weeks of the OCP injection in six of the cases. Five patients presented with symptoms post-OCP, and three were diagnosed asymptomatically on planned visits. Seven cases were phakic, one had high myopia (>8 dioptres), and two cases had lattice degeneration. Following RRD surgery, hole closure was achieved in 5/6 MH cases. The final median BCVA at 7 months was 20/80 (range 20/40–20/1200) similar to the baseline BCVA 20/80, with four patients gaining ≥1 line of vision compared to baseline but three losing ≥3 lines.

**Conclusions:**

RRD is a non-negligible risk associated with intravitreal OCP, and it should be used with caution in eyes with high myopia and peripheral retinal pathology predisposing to RRD. Detailed peripheral retinal examination is recommended pre- and postoperatively at all visits. Patients should be advised to seek attention if symptoms recur after initial presentation.

## Introduction

Ocriplasmin (OCP) (Jetrea, Thrombogenics, The Netherlands) has been licensed as a non-surgical treatment for symptomatic vitreomacular traction (VMT). It is a recombinant truncated form of human plasmin, and its chief mechanism of action is thought to be by the cleavage of peptide bonds in the vitreoretinal adhesion molecules laminin and fibronectin [1–3]. This action coupled with activation of endogenous matrix metalloproteinase-2 is thought to result in its ability to precipitate vitreoretinal separation [4]. Rhegmatogenous retinal detachment (RRD) has been reported after OCP injection, albeit as an infrequent occurrence, with an incidence of 0.4 % (2/465) of the OCP-treated patients in the pivotal microplasmin for intravitreous injection-traction release without surgical treatment (MIVI-TRUST) trials [5]. We report a series of eight RRD occurring after OCP injection, and describe their characteristics and outcomes following surgery.

## Method

In October 2014, the British and Eire Association of Vitreoretinal Surgeons (BEAVRS) organised an email survey of their members self-reporting their initial experiences with intravitreal OCP. The survey consisted of a short data-collection form on the number of patients treated with OCP, the indications for treatment, success rates in terms of VMT release and macular hole (MH) closure, as well as a list of possible observed adverse effects including retinal tears and detachments. Seven respondents reported eight patients with tears/retinal detachment, and these consultant vitreoretinal surgeons were then contacted with a more detailed data-collection questionnaire concerning their cases that are presented here. Snellen visual acuities were converted to logMAR for analysis. Under UK guidelines the study was classified as a service evaluation by the local ethics committee, and as such did not require formal ethical approval.

## Results

The initial email questionnaire collected data from a total of 241 OCP treated patients (241 eyes) by 41 surgeons. The indication for treatment was MH with vitreomacular adhesion (VMA) in 111 patients (46 %) and VMT alone in 130 (54 %). Eight patients were reported by seven surgeons with retinal tears and/or RRD following OCP. These surgeons were then sent a second questionnaire with a detailed data-collection form for each of the eight reported cases.

### Baseline findings prior to OCP injection

The mean age of the eight patients was 63 years old (range 51–76 years), and six (75 %) were female. The majority of cases were phakic (88 %) at the time of the procedure. The mean spherical equivalence was −3 dioptres (range −0.25 to −9 dioptres). The initial indication for OCP was VMT in two cases (25 %) and MH with VMA adhesion in six (75 %). The MH size was small (≤250 μm) in two eyes and medium (250–400 μm) in four eyes, with a mean minimum linear diameter of 276.8 μm (range, 192–380 μm). The mean duration of symptoms prior to OCP injection was 2 months (range, 0.75–10 months). The mean best-corrected visual acuity (BCVA) at baseline was 20/80 (range 20/40–20/400).

### Outcome summary

OCP was administered as per the manufacturer’s instructions by a single 0.1 ml intravitreal injection of 125 micrograms using a 30-g needle in all cases, with no immediate injection-related complications noted. Experienced retinal surgeons in specialized eye units carried out all injections. The RRD was diagnosed after a median of 16 days (range, 3–131 days) post-OCP injection. Five of the patients reported symptoms of photopsia starting within a few hours of injection and lasting up to 72 h. One patient presented with RRD during this phase on day 3. Four patients developed new symptoms of floaters and photopsia after this period, and presented symptomatically outside of scheduled appointments on days 6, 14, 18 and 20. Three patients were diagnosed asymptomatically at scheduled appointments on days 5 and 54 post-procedure, and day 131 at the time of planned vitrectomy surgery for a persistently open MH. In the 4/8 cases that were examined before presentation with RRD, VMA resolution was documented in two MH cases (25 %), and one (12.5 %) had developed a posterior vitreous detachment (PVD) from the optic disc head.

**Fig. 1:**
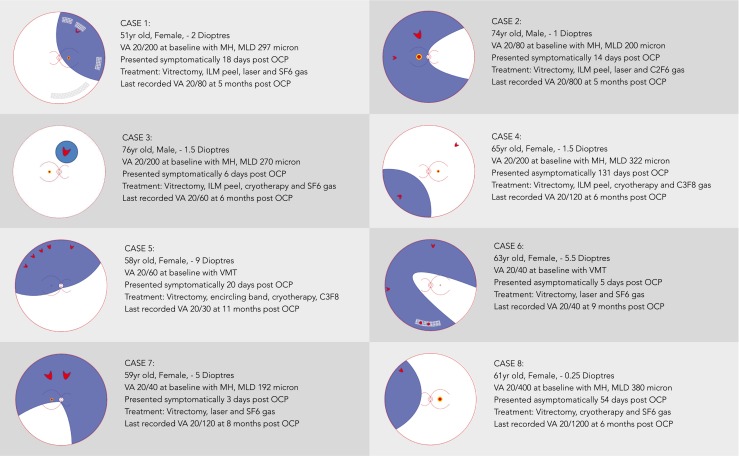
Diagrams of the rhegmatogenous retinal detachment cases showing associated retinal breaks and pathology

The findings at the time of RRD and surgical procedure performed are described in Fig. 1. Four of the cases were macula on and four macula off at presentation. At the time of the RRD repair, VMT resolution was noted in all cases, and PVD with a Weiss ring in 7/8 cases. All the cases had one or more superior tears. Cases 2 and 6 had additional tears at the 9 and 3 o’clock meridians respectively, and case 4 had an additional inferior tear and associated detachment. In cases 2, 3 and 7, the tears were located in the mid-periphery posterior to the equator, whilst in the other five cases the tears were more peripheral at the posterior border of the vitreous base between the equator and ora serrata. The distribution of breaks showed no predilection for any one quadrant (Chi square test, *p* = 0.38).

Cases 1 and 6 developed a re-detachment at 4 and 2 weeks post-primary RRD repair respectively. Case 1 was noted to have recurrent retinal detachment at 4 weeks post-initial RRD repair with early proliferative vitreoretinopathy infero-temporally, with a new retinal tear in the same area. Revision pars plana vitrectomy (PPV) combined with epiretinal membrane peeling, cryotherapy, and 25 % SF6 gas injection was performed successfully. At 6 months postoperatively the VA was 20/80, and retina was attached. Case 6 developed a total recurrent macula-off RRD with new tears at the 2-week follow-up visit after initial PPV, and required a revision procedure with laser and 14 % C3F8 gas. This case was then treated successfully for possible endophthalmitis 4 months later following uncomplicated cataract surgery with intravitreal antibiotics. The final VA at 14 months post RRD surgery and after cataract surgery was 20/40. Both of these cases had areas of lattice degeneration present.

All cases with MH had internal limiting membrane peeling performed at the time of vitrectomy surgery for the RRD; and following surgery, hole closure was achieved in 5/6 MH cases. The mean BCVA at 2 months post-OCP was 20/240 (range 20/40–20/1200). The final mean BCVA at a mean follow-up of 8 months (range 5–14 months) was 20/80 (range 20/40–20/1200), similar to the baseline BCVA. BCVA remained unchanged in one patient, and four patients gained ≥ 1 line in vision compared with pre -OCP BCVA. Three patients lost ≥ 3 lines in VA, although one of these cases was still awaiting cataract surgery at the time of data collection and subsequently died secondary to bowel cancer.s

## Discussion

We report the presentation and characteristics of eight cases of RRD following OCP, representing the largest series of cases to date. We are unable to give an incidence rate of RRD after OCP, as we do not know the true denominator of cases treated by members during the study period nor the completeness of case reporting to us. As well as the 0.4 % rate in the phase 3 studies of OCP [1], a single case (representing 1.7 %) was reported in the phase I MIVI study day 1 post-injection [3]. There have been reports of three cases of RRD [6–8] out of 213 [6–14] treated cases (1.4 %) in recent retrospective institutional studies, two of which occurred at day 6 and one at week 6 post-OCP injection.

The mean age of 72 years in the MIVI trials was similar to our cases. The majority of our cases were phakic (88 %) as opposed to 63 % in the MIVI OCP group. One of our cases had high myopia, which would have been excluded from the phase 3 MIVI trials, which had a cut off point of −8 dioptres of myopia. Two of the patients also had lattice degeneration. There have also been reports of two other patients with lattice degeneration who developed RRD after OCP in recent institutional reviews [7, 8]. This was not listed as an exclusion criterion in the phase II and III MIVI trials [1, 15] report but was in the phase I trials [3]. We would suggest that myopia greater than 8 dioptres and lattice degeneration should be considered as contraindications to OCP treatment. Whether treating lattice preoperatively would reduce the risk of RRD is unknown.

The median time to onset of RRD was 16 days after OCP injection, with 37.5 % presenting within the first 7 days and the rest presenting on days 14, 18, 20, 54, and 131. Photopsia and floaters are common symptoms in the first few days after OCP injection, and consistent with the action of the drug [5]. One of our cases presented whilst still symptomatic with photopsia in the immediate postoperative period, while in four cases there was recurrence of symptoms after the postoperative symptoms had settled. Three of our cases, however, were diagnosed whilst asymptomatic at routine review appointments. Our cases show that RRD post-OCP can be asymptomatic, whilst others with symptoms may be difficult to distinguish from typical post OCP symptoms. We recommend that scheduled appointments at the peak time of RRD in the first few postoperative weeks, as well as careful peripheral retinal examination at all visits, is important even if asymptomatic.

There has been some speculation that breaks occurring soon after ocriplasmin may have been a result of the physical action of the intravitreal OCP injection in yielding vitreoretinal traction [6]. This is supported by the fact that during the repair of the retinal tear and detachment in the MIVI I trials, the vitreous was found still firmly adherent to the retina [3]. In our series, 3/7 presented with RRD within 7 days post-injection; however, five cases presented 14–130 days post-OCP. Given the timing of our five cases and one reported case in the literature at 6 weeks post-OCP injection [7], and the fact that VMT had released in all cases, makes OCP’s pharmacological action the likely culprit, rather than the physical action of the intravitreal injection. It is important to note that although 26.5 % of OCP-injected eyes had vitreo-foveal separation in the MIVI TRUST trials at day 28, complete PVD was noted in only 13.4 % of eyes at the same time point. It is possible, therefore, that more peripheral vitreous separation occurs later in OCP-treated eyes, and the true incidence of tear and RRD formation may ultimately be higher than during the limited follow-up period carried out during the phase 3 studies. We found complete PVD at the time of surgery in 88 % of cases, and it’s clearly important to maintain vigilance for late complete vitreous separation and consequent retinal tears.

Although OCP can induce a clean plane of vitreoretinal separation [2, 16, 17] it does not appear to do this uniformly across the whole VR interface. In a study characterising the effect of OCP on the vitreoretinal interface in an ex-vivo porcine model, the vitreolytic effect was not found to be homogenous throughout the eye [18]. Retinal areas proximal to the site of injection in the mid vitreous frequently appeared to be more devoid of vitreous elements in comparison to more anterior areas. The authors concluded that although this may in part reflect a difference in chemical structure of the vitreous adjacent to the vitreous base, it might reflect a lack of exposure to the enzyme that has to diffuse further to this particular location from its site of injection. This raises the question as to whether other factors, such as variable diffusion through the vitreous and variations in drug preparation and injection technique, might also influence the effect of the drug with its short half-life [19]. The distribution of retinal breaks would also concur with this, in that the breaks were not concentrated in the superotemporal quadrant as found in spontaneous PVD-related tears and RRD [20]. Spontaneous PVD is thought to occur perifoveally and extends first superiorly [21]. Surgically induced vitreous separation results in a different retinal break distribution to spontaneous PVD, with a more evenly distributed tear distribution or possibly a higher incidence of tears inferiorly [22–24]. It is debated whether intravitreal injection of OCP should be performed with more pressure or deeper into the vitreous cavity than vascular endothelial growth factor inhibitors [12]. In our series, OCP was administered according to the label, without interruption of the freezing cycle and using a standard 30-gauge needle. The depth and site of injection was not recorded, but no complications immediately following injection were noted. It is possible that OCP-mediated vitreous liquefaction can result in an incomplete PVD leaving residual vitreous cortex attached to the retina peripherally, ultimately contributing to late retinal break formation [25].

We asked that surgeons report all cases of retinal tears and RRD to us, but interestingly there were no cases of tears without RRD reported. This is unusual, as it is known that not all tears associated with spontaneous vitreous separation progress to RRD [26]. There have been many reports of subretinal fluid accumulation at the fovea after OCP, and Willekens et al. reported this in 37 % of cases treated including peripapillary SRF in some cases [7]. Increase in MH base diameter has also been widely reported [5, 14, 27]. The aetiology of this is unknown. There is plausible evidence that OCP causes weakening of retinal adhesion by degrading laminin and possibly other proteins in the outer retinal layers including the interphotoreceptor matrix [28], which is known to mediate retinal pigment epithelial-photoreceptor adhesion in primate eyes [29]. This mechanism might also mean that tear formation has a higher incidence of progression to RRD after OCP than after spontaneous PVD. Interestingly, in Pierson syndrome, which is caused by mutations in the laminin b2 gene, the incidence of RRD is higher [30]. This provides further evidence that laminin function is important for maintaining retinal attachment, and perhaps tears induced after PVD from OCP are more prone to progress to RRD.

There are several weaknesses to this study, in particular the absence of a clear denominator number for the number of cases treated in total with OCP that subsequently resulted in the eight retinal detachments. We therefore do not know the true incidence of RRD after OCP in our population. The study was retrospective, and the follow-up after injection was at the discretion of the treating specialist. There is therefore some uncertainty of the timing of retinal detachment in some of the cases.

## Conclusion

RRD can occur after OCP injection, and patients should be counselled preoperatively regarding the non-negligible risk of RRD. We would recommend that OCP is used with caution in eyes with greater than 8 dioptres of myopia and peripheral retinal pathology predisposing to RRD, including lattice degeneration. We would recommend that clinicians perform detailed peripheral retinal inspections preoperatively and at planned regular post-injection clinic visits. Furthermore, patients should be advised to seek attention if symptoms recur after the immediate perioperative period. It is possible that the real-world incidence of RRD with less strict exclusion criteria and longer follow-up will be higher than in the phase 3 trials, and a further ongoing study is needed.
